# Clinical outcomes following long versus short cephalomedullary devices for fixation of extracapsular hip fractures: a systematic review and meta-analysis

**DOI:** 10.1038/s41598-021-03210-1

**Published:** 2021-12-14

**Authors:** Gabriel Kai Yang Tan, Christoph Sheng Chong, Hamid Rahmatullah Bin Abd Razak

**Affiliations:** 1 Lee Kong Chian School of Medicine, Nanyang Technological University, Singapore, 11 Mandalay Road, Singapore, 308232 Singapore; 2 Department of Orthopaedic Surgery, Sengkang General Hospital, 110 Sengkang East Way, Singapore, 544886 Singapore; 3 SingHealth Duke-NUS Musculoskeletal Sciences Academic Clinical Programme, Academia Level 4, 20 College Road, Singapore, 169856 Singapore

**Keywords:** Outcomes research, Trauma

## Abstract

Although both long and short cephalomedullary devices (CMDs) are used in the treatment of extracapsular hip fractures, the advantages of either option are subject to debate. This study aims to evaluate the differences in clinical outcomes with long versus short CMDs for extracapsular hip fractures. Studies included must have included subjects with at least 1 year of follow-up and reported on at least one of the following outcomes: rate of reoperation; rate of peri-implant fracture; operating time; blood loss; complication rate; length of hospital stay; 1-year mortality. Only articles written in the English language were included in this study. A search was conducted across the databases of Medline, Embase, CENTRAL (Cochrane Central Register of Controlled Trials), CINAHL and Scopus for articles published from the inception of the database to 1 November 2020. Included studies were assessed for their risk of bias using the Risk of Bias Tool (RoB2) and the risk-of-bias in non-randomized studies – of interventions (ROBINS-I) tool. A total of 8460 fractures from 16 studies were included in the analysis, with 3690 fixed with short, and 4770 fixed with long CMDs. A meta-analysis of the results revealed that short CMDs offer peri-operative advantages, while long CMDs could offer longer-term advantages. Limitations of this study include a lack of randomized control trials included in the analysis. In conclusion, when planning for the treatment of extracapsular hip fractures, a patient specific approach may be necessary to make a decision according to the individual risk profile of the patient.

## Introduction

Hip fractures are known to be increasing in frequency globally due to an ageing and active global population, with annual incidences set to rise to 6.26 million by 2050^1^. Furthermore, with a 1-year mortality rate of about 20%, being able to choose the optimal treatment of a hip fracture is of increasing importance and relevance^2,3^. Of the different types of hip fractures, extracapsular fractures such as basicervical neck of femur fractures and intertrochanteric (IT) fractures are thought to be the most associated with falls from a standing height in elderly patients who have osteoporosis^4^.

Currently there is an increasing trend towards the using a cephalomedullary device (CMD) to manage both stable and unstable extracapsular hip fractures (EHFs) as these nails act as an intramedullary buttress to prevent excessive shaft medialization^5–7^. When compared with previous surgical options for management, CMDs have shown more favourable long term outcomes and a lower rate of complications^8^. CMDs which are < 250 mm in length are generally considered to be short, while those longer than 250 mm are classified as long CMDs, the difference being short CMDs do not cross the isthmus of the femur^9^.

Previous studies have compared the biomechanical properties of long and short CMDs and their effectiveness in fixation of EHFs. While it has been found that axial stiffness is greater in the use of short CMDs, the overall results show no significant differences between short or long CMDs and suggest that either option can be employed for fixation of unstable EHFs^10,11^.

Although both long and short CMDs are used in the treatment of EHFs, the theoretical advantages of either option are subject to debate^12^. In recent years, research into this topic has shown advantages of short CMDs over long CMDs, with better intra-operative outcomes, including a shorter operative time, less blood loss and lower rate of transfusion^13,14^. Conversely, there are also advantages for the use of long CMDs over short CMDs. The longer nail length theoretically provides increased stability in unstable patterns due to the possible distal subtrochanteric extension^10,15^. There have also been lower reported rates on the incidence of peri-implant fractures, possibly due to the full-length nail providing protection to the entire femoral shaft^16,17^. But these differences have not affected long term outcomes in patients, with both groups showing no significant difference in reoperation rates, complication rates or 1-year mortality rates^18,19^.

However, these findings have not been clearly shown in large scale review papers and meta-analyses. This systematic review and meta-analysis of current literature aims to evaluate the differences in clinical outcomes with long versus short cephalomedullary devices (CMDs) for extracapsular hip fractures with the primary outcome being operative time and secondary outcomes being complications such as blood loss and peri-implant fractures.

## Methods

The systematic review was planned, conducted, and reported according to the PRISMA guidelines.

### Eligibility criteria

To be included in this study, articles had to be either randomized controlled trials, retrospective or prospective cohort studies. These studies must have also reported on simple or multifragmentary EHFs (AO classification 31-A1, A2, and A3) and compared results from patients treated with long CMDs versus short CMDs. The studies must have also had subjects with at least 1 year of follow up, and reported on at least one of the following outcomes, operating time; estimated blood loss; length of hospital stay; overall rate of peri-implant fracture; overall rate of reoperation; overall complication rate; 1-year mortality. Only articles written in the English language were included in this study.

**Figure 1:**
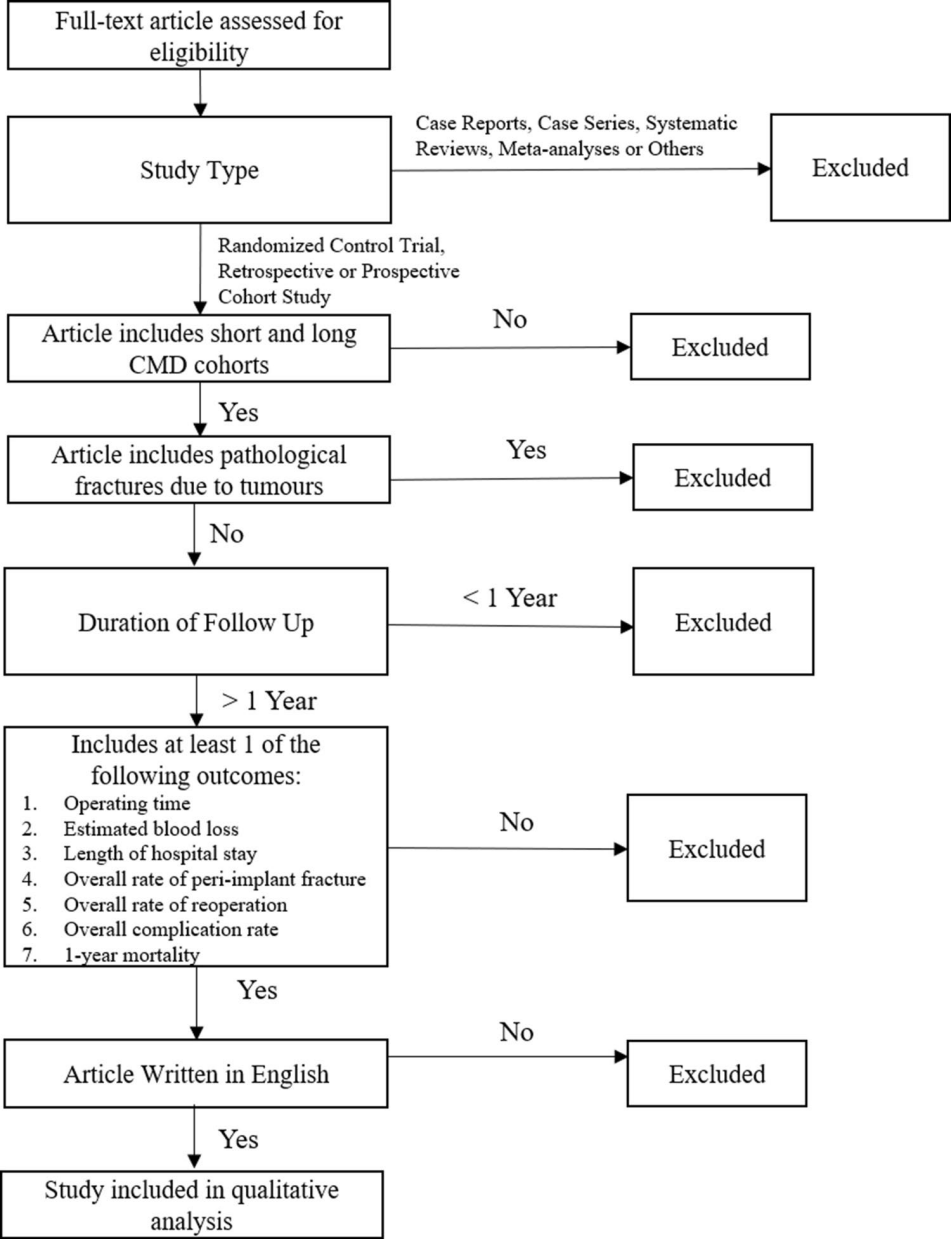
Eligibility criteria.

Case reports, case series, systematic reviews and meta-analyses were excluded from this study. Additionally, articles which did not include both short and long CMD cohorts for comparison, had less than 1 year follow up, or included pathological fractures due to tumours were also excluded (Fig. 1).

### Search strategy

A systematic search was conducted across the databases of Medline, Embase, CENTRAL (Cochrane Central Register of Controlled Trials), CINAHL and Scopus for articles published from the inception of the database to 1 November 2020. The search strategy used was based on: (Hip Fractures or Intertrochanteric Fractures or pertrochanteric fracture*) and (Fracture Fixation, Intramedullary or cephalomedullary nail* or cephalomedullary nail* or intermedullary nail*).

### Study selection

Duplicate studies were identified and removed by Covidence, a systematic review tool which uses the Cochrane technology platform. Two independent reviewers then screened the identified studies for relevance. Full manuscripts of included studies were assessed according to the eligibility criteria and the data was extracted. Any inconsistency was resolved through discussion between the two reviewers.

### Statistical analysis

All statistical analyses were performed using Cochrane Revman 5.3 Software (Cochrane Collaboration 2014). Risk ratios and 95% confidence intervals were calculated. A *p* value of < 0.05 was considered significant for this study.

## Results

### Systematic review

#### Study selection

**Figure 2:**
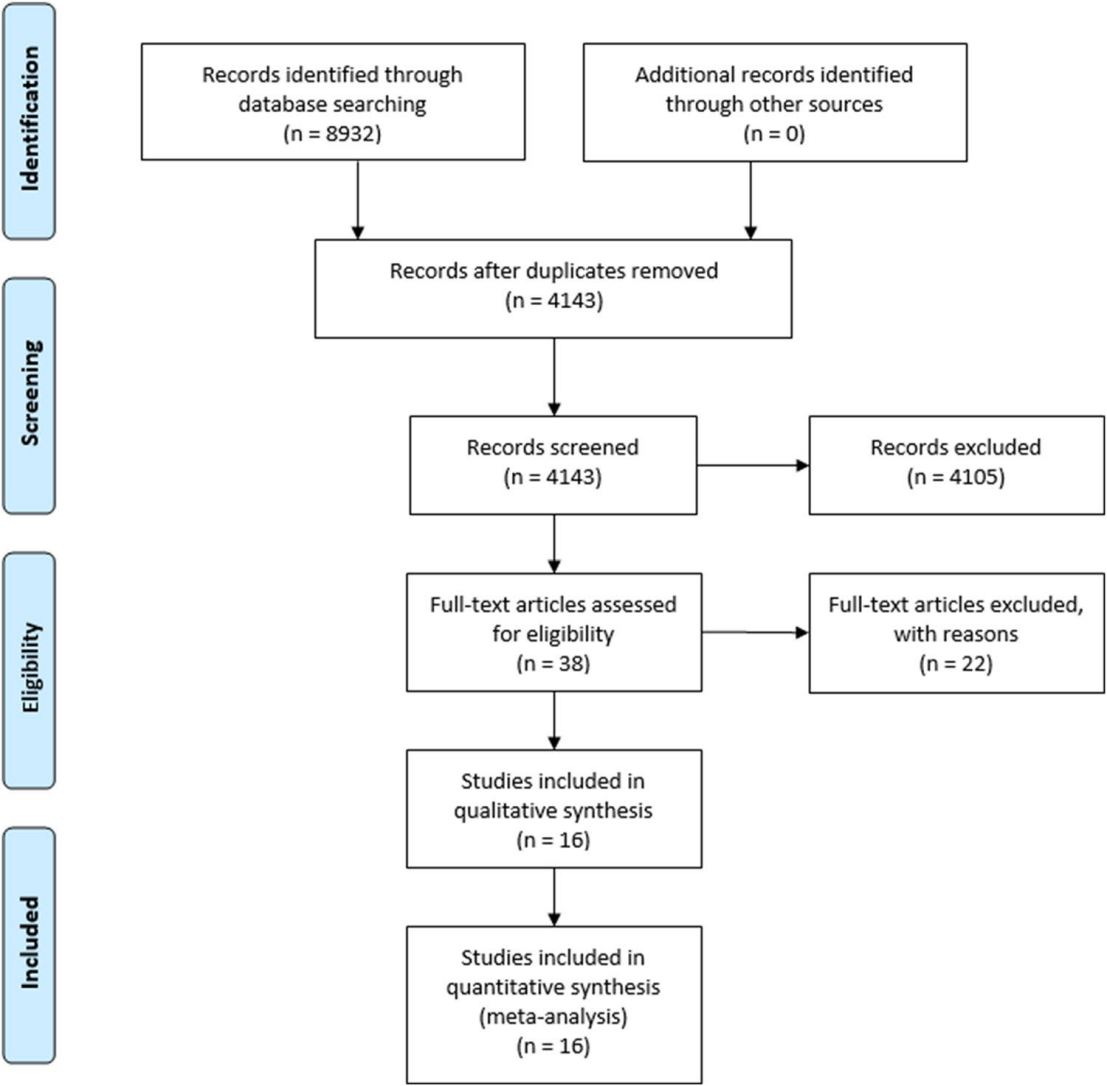
Flowchart of studies in review.

A total of 4143 studies were identified through the database searches, with 16 meeting the inclusion criteria and being included in this study (Fig. 2). A total of 8460 fractures were included in the analysis, with 3690 fixed with short, and 4770 fixed with long CMDs. The study with the largest sample size was also the most recently published, by Sadeghi et al*.* with 5526 patients^20^. Detailed information on the studies included are included in Table 1.

**Table 1:** Eligible studies, study characteristics, population demographics.

S/N	Study	Country	Inclusion period	Study design	Device model (short/long)	Number of fractures	Sample size (short/long)		Gender (M/F)	Mean age/years (SD)	Length of F/u (months)	ASA score (1/2/3/4)	OTA classification (31A1/2/3)
1	Hulet et al.^21^	USA	2000–2012	Retrospective cohort	NI	201	70	131	Short 23/47 Long 57/74 (*p* = 0.140)	Short 70.6 (19.1) Long 70.1 (17.4) (*p* = 0.640)	40.6 (range 6–158)	NI	Short 23/28/19 Long 19/57/55 (*p* = 0.010*)
2	Krigbaum et al.^22^	USA	2001–2010	Retrospective cohort	NI	262	125	137	Short 120/5 (*p* = 0.880)	(*p* = 0.340)	Long 33.6 (SD 31.2) Short 24 (SD 26.4)	NI	Short 0/125/0
3	Kleweno et al.^23^	USA	2004–2010	Retrospective cohort	Gamma 3, Synthes TFNA/Gamma 2, Gamma 3, Synthes TFNA	559	219	340	NI	NI	30.1 (range 12–85)	NI	NI
4	Hou et al.^18^	China	2005–2009	Retrospective cohort	TFNA (170 mm)/TFNA	283	100	183	Short 16/84 Long 57/126 (*p* = 0.006*)	Short 81.0 (range 53–102) Long 78.6 (range 47–98) (*p* = 0.064)	37 (SD 2.3)	Short 0/11/64/14 (NI = 11) Long 1/24/101/32 (NI = 25)	Short 59/41/0 Long 67/116/0 (*p* = 0.000*)
5	Frisch et al.^24^	USA	2005–2010	Retrospective cohort	InterTAN/InterTAN	169	72	97	Short 18/54 Long 30/67 (*p* = 0.621)	Short 76.2 (12.3) Long 76.3 (15.2) (*p* = 0.501)	NC	NI	NI
6	Vaughn et al.^12^	USA	2006–2011	Retrospective cohort	Gamma 3/Gamma 3	256	60	196	NI	NI	> 12	NI	Short 37/23/0 Long 106/90/0
7	Boone et al.^25^	USA	2008–2011	Retrospective cohort	Gamma 3/Gamma 3	201	82	119	Short 25/57 Long 32/87 (*p* = 0.578)	Short 83.3 (8.0) Long 79.6 (9.6) (*p* = 0.005*)	NC	NI	Short 31/51/0 Long 28/91/0 (*p* = 0.029)
8	Guo et al.^26^	China	2008–2013	Retrospective cohort	Gamma 3 (180 mm)/Gamma 3 (320, 340, 360 mm)	178	102	76	Short 42/60 Long 43/33 (*p* = 0.322)	Short 82.7 (9.9) Long 78.9 (8.8) (*p* = 0.003*)	23.1 (SD 6.8)	NI	Short 47/55/0 Long 26/50/0 (*p* = 0.037)
9	Sellan et al.^27^	Canada	2008–2013	RCT	InterTAN (180–200 mm)/InterTAN (260–460 mm)	108	71	37	Short 20/51 Long 14/23 (*p* = 0.259)	Short 80.6 (0.9) Long 78.0 (1.7) (*p* = 0.171)	> 12	NI	All A1/A2
10	Okcu et al.^28^	Turkey	2009	RCT	PFNA (240 mm)/PFNA (340–420 mm)	33	15	18	Short 4/11 Long 4/14 (*p* = 0.767)	Short 78 (range 67–95) Long 81 (range 73–89) (*p* = 0.255)	Short 14.0 (range 12–19) Long 14.5 (range 12–21)	NI	Short 0/0/15 Long 0/0/18
11	Hong et al.^29^	Singapore	2009–2012	Retrospective cohort	PFNA (200 mm)/PFNA (320, 340, 380 mm)	64	44	20	Short 13/31 Long 6/14 (*p* = 0.971)	Short 80.0 (range 60–93) Long 79.8 (range 56–97) (*p* = 0.920)	> 12	Short 1/22/21/0 Long 1/9/10/0 (*p* = 0.813)	Short 11/33/0 Long 4/16/0 (*p* = 0.662)
12	Sadeghi et al.^20^	USA	2009–2014	Retrospective cohort	Gamma 3 (170–180 mm), TFNA (170–180 mm)/Gamma 3, TFNA	5526	2418	3108	Short 720/1698 Long 938/2170	Short 81.2 (10.8) Long 80.6 (11.0)	NC	Short (mean = 2.9) Long (mean = 2.8) (*p* = 0.600)	NI
13	Li et al.^30^	China	2010–2012	Retrospective cohort	PFNA/PFNA	156	97	59	Short 46/51 Long 20/39 (*p* = 0.116)	Short 76.81 (6.56) Long 74.85 (8.15) (*p* = 0.100)	NC	Short 49/31/18 Long 29/19/11 (*p* = 0.210)	Short 17/27/15 Long 28/44/25 (*p* = 0.102)
14	Raval et al.^31^	UK	2011–2012	Retrospective cohort	PFNA (240 mm) /PFNA (340–400 mm)	80	40	40	Short 11/29 Long 13/27 (*p* = 0.625)	Short 77.1 (9.2) Long 76.1 (8.7) (*p* = 0.806)	> 12	NI	Short 12/24/4 Long 9/24/7 (*p* = 0.536)
15	Bovbjerg et al.^32^	Denmark	2012	Retrospective cohort	Gamma 3/Gamma 3	216	95	121	Short 28/67 Long 43/78 (*p* = 0.346)	Short 83.1 (8.35) Long 82.9 (7.69) (*p* = 0.884)	> 12	NI	NI
16	Shannon et al.^33^	USA	2014–2017	RCT	Synthes TFNA, Gamma 3, Affixus/ Synthes TFNA, Gamma 3, Affixus	168	80	88	Short 20/60 Long 25/63 (*p* = 0.720)	Short 82 (range 79–84) Long 79 (range 76–82) (*p* = 0.110)	Short 10.5 Long 10.2	NI	Short 13/61/6 Long 12/67/9 (*p* = 0.230)

*RCT* randomized controlled trial, *NI* no information, *NC* not possible to calculate.

*Indicates statistically significant difference reported.

All included studies were subject to an assessment of bias, with the revised Cochrane risk-of-bias tool (RoB 2) for the randomized controlled trials, and Risk-of-bias in Non-randomized Studies—of Interventions (ROBINS-I) tool for the retrospective cohort studies^34,35^. The detailed information on the assessment for bias is reported in Tables 2 and 3 respectively. None of the studies received external funding.

**Table 2:** Intra-operative results.

Study		Mean operating time/min (SD/range)	*p* value	Mean est. blood loss/mL (SD/range)	*p* value	No. of patients requiring transfusion (%)	*p* value	Length of stay/days (SD/range)	*p* value
Hulet et al.^21^	Short	NI	NI	NI	NI	NI	NI	NI	NI
	Long	NI		NI		NI		NI	
Krigbaum et al.^22^	Short	66 (30.00)	< 0.001*	NI	NI	NI	NI	6.9 (4.8)	0.018*
	Long	90 (48.00)		NI		NI		9.1 (8.9)	
Kleweno et al.^23^	Short	51 (22.00)	< 0.00*	NI	NI	NI	NI	NI	NI
	Long	70 (35.00)		NI		NI		NI	
Hou et al.^18^	Short	41 (range 19–106)	< 0.000*	100	0.031*	42 (42.0%)	0.462	6.4	0.383
	Long	61 (range 16–216)		135		83 (45.4%)		6.8	
Frisch et al.^24^	Short	63.8 (20.00)	0.001*	Long 161.4 (122.40)	0.002*	NI	NI	NI	NI
	Long	82.6 (26.40)		Short 208.1 (116.90)		NI		NI	
Vaughn et al.^12^	Short	NI	NI	NI	NI	NI	NI	NI	NI
	Long	NI		NI		NI		NI	
Boone et al.^25^	Short	44.0 (10.70)	< 0.001*	92.6 (47.20)	0.002*	33 (40.2%)	0.002*	7.7 (4.10)	0.393
	Long	56.8 (19.40)		135.5 (91.90)		68 (57.1%)		8.0 (4.50)	
Guo et al.^26^	Short	43.5 (12.30)	0.002*	90.7 (50.60)	0.004*	42.3%	0.041*	12.9 (6.50)	0.420
	Long	58.5 (20.30)		127.8 (85.90)		56.7%		12.7 (6.20)	
Sellan et al.^27^	Short	60 (range 30–120)	0.021*	NI	NI	33 (46.4%)	0.364	20.2 (2.80)	0.345
	Long	73 (range 30–203)		NI		16 (41.0%)		15.7 (3.70)	
Okcu et al.^28^	Short	52.6 (range 34–65)	< 0.001*	NI	NI	NI	NI	5.4 (range 2–11)	0.510
	Long	71.8 (range 57–94)		NI		NI		4.9 (range 2–9)	
Hong et al.^29^	Short	73 (range 40–121)	0.617	NI	NI	NI	NI	15.5 (range 4–53)	0.793
	Long	78.2 (range 29–315)		NI		NI		14.0 (range 3–30)	
Sadeghi et al.^20^	Short	47.4 (22.80)	NI	99.8 (105.50)	NI	NI	NI	5.34 (4.24)	NI
	Long	62.7 (33.10)		135.7 (151.70)		NI		5.57 (4.43)	
Li et al.^30^	Short	53.08 (8.51)	0.000*	69.95 (21.55)	0.063	NI	NI	NI	NI
	Long	60.61 (11.43)		77.97 (31.88)		NI		NI	
Raval et al.^31^	Short	58.6 (12.60)	0.016*	Long 172.7 (156.90)	0.042*	4 (10.0%)	0.210	11.1 (6.20)	0.937
	Long	87.7 (32.60)		Short 341.7 (191.80)		8 (20.0%)		10.9 (4.80)	
Bovbjerg et al.^32^	Short	NI	NI	NI	NI	NI	NI	NI	NI
	Long	NI		NI		NI		NI	
Shannon et al.^33^	Short	51 (range 48–55)	< 0.0001*	70 (range 61–79)	< 0.001*	NI	NI	NI	NI
	Long	80 (range 74–87)		207 (range 185–229)		NI		NI	

*NI* no information.

*Indicates statistically significant difference reported.

**Table 3:** Post-operative results and complications.

Study		1-Year post-operative mortality (%)	*p* value	Overall complication rate (%)	*p* value	Overall rate of reoperation	*p* value	Overall rate of peri-prosthetic fractures	*p* value	Overall rate of peri-prosthetic infections	*p* value	1 Year post-operative harris hip score (SD)	*p* value
Hulet et al.^21^	Short	33 (47.14%)	NI	19 (27.14%)	NI	NI	NI	0 (0.00%)	NI	NI	NI	NI	NI
	Long	41 (31.30%)		34 (25.95%)		NI		0 (0.00%)		NI		NI	
Krigbaum et al.^22^	Short	35 (28%)	0.33	21.0%	0.710	5.00%	0.120	NI	NI	NI	NI	NI	NI
	Long	47 (34%)		19.0%		1.00%		NI		NI		NI	
Kleweno et al.^23^	Short	NI	NI	80 (36.5%)	0.930	7 (3.20%)	0.810	6 (2.70%)	0.35	NI	NI	NI	NI
	Long	NI		122 (35.9%)		12 (3.50%)		5 (1.50%)		NI		NI	
Hou et al.^18^	Short	22 (22.0%)	0.785	10 (10.0%)	0.518	5 (5.0%)	0.809	0 (0.00%)	0.178	1 (1.00%)	0.942	NI	NI
	Long	42 (23.0%)		23 (12.6%)		8 (4.4%)		2 (1.10%)		2 (1.10%)		NI	
Frisch et al.^24^	Short	NI	NI	NI	NI	NI	NI	6 (8.30%)	0.013*	1 (1.40%)	0.637	NI	NI
	Long	NI		NI		NI		0 (0.00%)		3 (3.10)		NI	
Vaughn et al.^12^	Short	NI	NI	4 (6.63%)	NI	NI	NI	2 (3.33%)	NI	NI	NI	NI	NI
	Long	NI		9 (4.60%)		NI		0 (0.00%)		NI		NI	
Boone et al.^25^	Short	NI	NI	NI	NI	NI	NI	1 (0.84%)	NI	NI	NI	NI	NI
	Long	NI		NI		NI		0 (0.00%)		NI		NI	
Guo et al.^26^	Short	NI	NI	3	> 0.05	NI	NI	1 (0.90%)	NI	1 (0.90%)	> 0.05	NI	NI
	Long	NI		4		NI		1 (1.30%)		1 (1.30%)		NI	
Sellan et al.^27^	Short	11 (15.5%)	0.783	39	NI	NI	NI	5 (7.00%)	0.35	NI	NI	NI	NI
	Long	5 (12.8%)		27		NI		1 (2.60%)		NI		NI	
Okcu et al.^28^	Short	3 (16.6%)	0.9	3 (20.0%)	0.390	0	0.410	NI	NI	NI	NI	74 (8)	0.11
	Long	5 (18.1%)		6 (33.3%)		2		NI		NI		79 (10)	
Hong et al.^29^	Short	2 (4.50%)	0.625	NI	NI	4 (9.10%)	0.689	3 (6.80%)	NI	NI	NI	NI	NI
	Long	0 (0.00%)		NI		1 (5.00%)		0 (0.00%)		NI		NI	
Sadeghi et al.^20^	Short	NI	NI	NI	NI	46 (1.90%)	NI	14 (0.60%)	NI	1 (2.20%)	NI	NI	NI
	Long	NI		NI		50 (1.60%)		13 (0.40%)		0 (0.00%)		NI	
Li et al.^30^	Short	NI	NI	3 (3.00%)	< 0.05*	NI	NI	NI	NI	NI	NI	76.16 (10.84)	0.28
	Long	NI		0 (0.00%)		NI		NI		NI		79.98 (8.9)	
Raval et al.^31^	Short	3 (7.50%)	0.456	NI	NI	1 (2.50%)	0.556	NI	NI	NI	NI	NI	NI
	Long	5 (12.50%)		NI		2 (5.00%)		NI		NI		NI	
Bovbjerg et al.^32^	Short	NI	NI	5 (5.25%)	NI	NI	NI	1 (1.05%)	NI	0 (0.00%)	NI	NI	NI
	Long	NI		7 (5.80%)		NI		1 (0.83%)		0 (0.00%)		NI	
Shannon et al.^33^	Short	NI	NI	12 (15.5%)	0.830	5 (6.25%)	0.720	2 (2.49%)	1	1 (1.25%)	1	76 (3 months) (IQR 74–78)	0.02*
	Long	NI		12 (13.60%)		8 (9.09%)		2 (2.27%)		2 (2.27%)		71 (3 months) (IQR 68,074)	

*NI* no information.

*Indicates statistically significant difference reported.

#### Study characteristics

Three studies, Sellan et al*.*, Okcu et al*.* and Shannon et al*.*, had a randomised controlled trail (RCT) design^27,28,33^. The remaining thirteen studies were retrospective cohort studies. Half of the studies were performed in the USA, with the remaining from Europe and Asia.

#### Device models

Data on the device models were collected from most studies, demonstrating a wide spectrum of device preferences across the board. Majority of the studies used a fixed device brand with nails of varying length for comparison, with the notable exception of Kleweno et al*.*, which opted to include the Gamma 2 nail as a choice of long nail despite not including it under the short nail devices^23^.

#### Sample population characteristics

Most of the included studies had largely similar demographics of gender and age. However, Hou et al*.* noted a significant preponderance of females over males^18^. Boone et al*.* and Guo et al*.* also included significant differences in the ages of patients offered short and long nails, with older patients tending towards a short nail^25,26^. The included studies have a range of mean follow-up duration of 10.2–40.6 months post-operatively. Additionally, each study had at least one patient who was followed up for a minimum of 12 months post-operatively. ASA score was reported in four studies, ranging from 1 to 4^18,20,29,30^.

#### Fracture pattern

Twelve studies reported on the fracture classification, with ten further subclassifying the fractures under the length of nail used. Many (n = 6) focused on 31A1/2 fractures, but some studies (n = 4) also elected to include patients from all 31A fractures. Of note, Okcu et al*.* only studied patients with 31A3 fractures^28^.

#### Operative time

Twelve studies included data on operative times for the surgical procedures. All but one study reporting a significantly longer operating time for long CMDs as compared to short CMDs, with Hong being the only dissenting study^18,22–31,33^.

#### Blood loss

Eight studies reported on the estimated blood loss from either procedure, with 7 studies reporting statistically significantly higher mean estimated blood loss in long CMD operations as opposed to short CMDs^20,24–26,30,31,33^. Five studies included information on the number of patients requiring blood transfusion, however only Boone et al*.* reported that there was a statistically significant difference in transfusion rates^18,25–27,31^.

#### Length of stay (LOS)

A total of 9 studies included the LOS of patients post-operatively, with 8 of the studies reporting no significant difference in LOS between the two groups^18,20,22,25–29,31^.

Information on intraoperative results have been reported in Table 2.

#### Post-operative results and complications

Seven studies reported on the 1-year mortality rates of patients, with none of them finding any significant difference between the two groups^18,21,22,27–29,31^.

1-year complication rate was reported in 11 studies, with only Li et al*.* reporting statistically fewer incidences in the long CMD group^12,18,21–23,26–28,30,32,33^.

The 1-year reoperation rate was reported to be statistically similar in the 8 studies which included the information^18,20,22,23,28,29,31,33^.

Peri-implant fracture rates 1-year post-operatively were reported in 10 studies, with only Frisch et al. reporting a statistically higher rate of fractures on the short CMD group^12,18,20,21,23,24,26,27,29,32,33^.

Peri-implant infection rates 1-year post-operatively were also reported to be similar in the 6 studies which reported on them^18,20,24,26,32,33^.

Only three studies noted patient reported outcomes under the Harris Hip Score 1-year post-operatively. Of the studies, only Shannon et al*.* reported a significant difference between the groups, with the short CMD group reporting higher results^28,30,33^.

Detailed information on post-operative results and complications can be found in Table 3.

### Meta-analysis

We performed a meta-analysis to compare several outcomes of interest between long and short CMD groups. The outcomes analysed were mean operating time, mean estimated blood loss, mean length of stay, peri-implant fracture rates, reoperation rates and 1-year mortality rates. All 16 included studies were included in the meta-analysis.

#### Mean operating time

**Figure 3:**
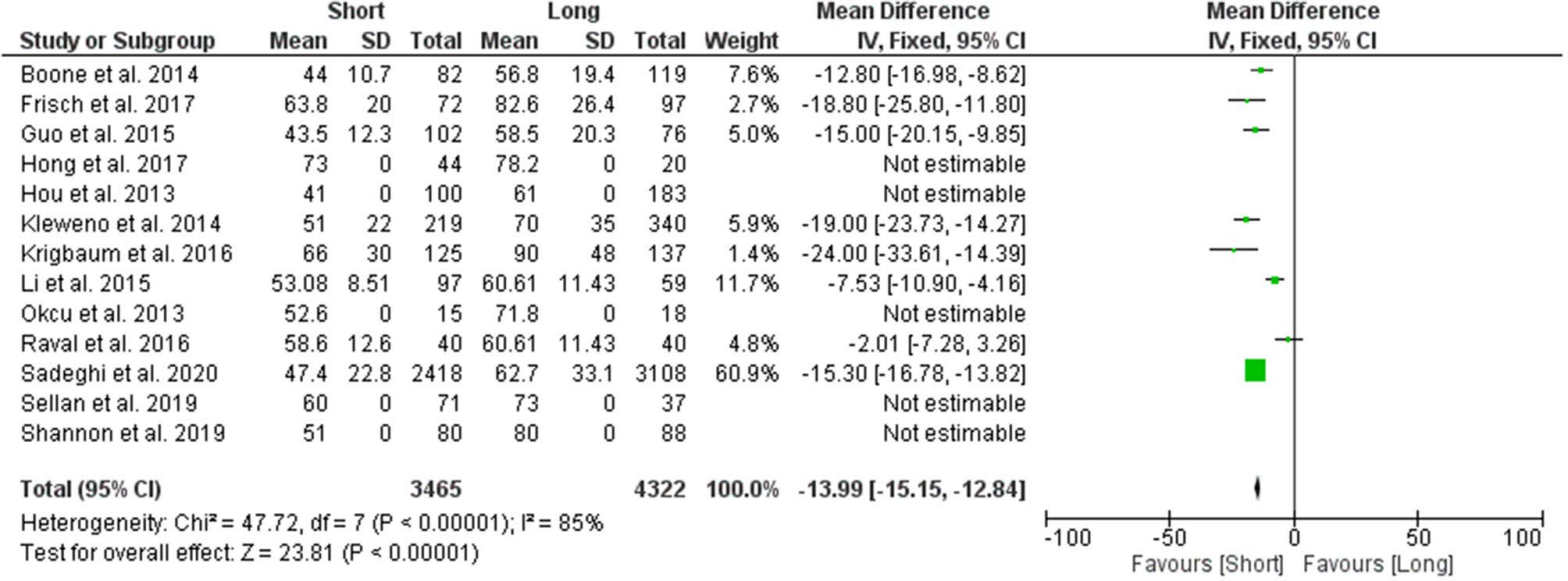
Forest plot of mean operating times between long and short CMD groups. Green boxes represent weighted mean values of each study. The black diamond represents the overall pooled weighted mean of all included studies.

A total of 8 studies were used to analyse the difference in mean operating time between the two groups. Results from the analysis favoured the group which used short CMDs, reporting a statistically significant lower mean operating time of 13.99 min (95% CI − 15.15 to − 12.84; *p* value < 0.00001) (Fig. 3).

#### Mean estimated blood loss

**Figure 4:**
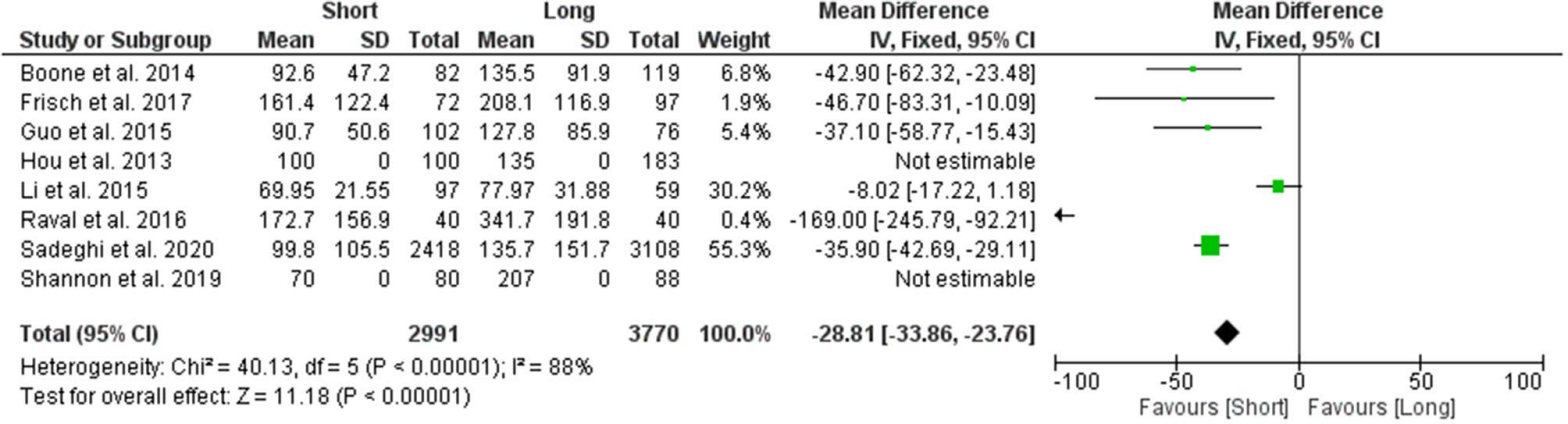
Forest plot of mean estimated blood loss between long and short CMD groups. Green boxes represent weighted mean values of each study. The black diamond represents the overall pooled weighted mean of all included studies.

A total of 6 studies were used to analyse the difference in mean estimated blood loss between the two groups. Results from the analysis favoured the group which used short CMDs, reporting a statistically significant lower estimated blood loss with a difference of 28.81 mL (95% CI − 33.86 to − 23.76; *p* value < 0.00001) (Fig. 4).

#### Mean length of stay

**Figure 5:**
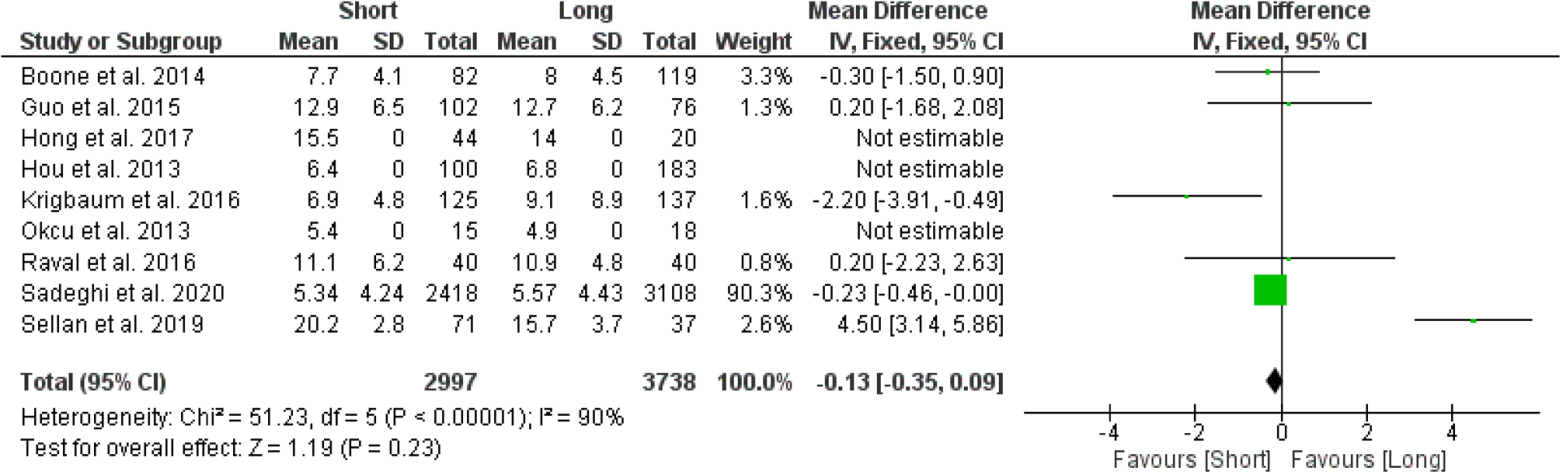
Forest plot of mean length of stay between long and short CMD groups. Green boxes represent weighted mean values of each study. The black diamond represents the overall pooled weighted mean of all included studies.

A total of 6 studies were used to analyse the difference in mean length of stay between the two groups. Results from the analysis favoured the group which used short CMDs, however this difference was found to not be statistically significant (95% CI − 0.35 to 0.09; *p* value = 0.23) (Fig. 5).

#### Peri-implant fractures

**Figure 6:**
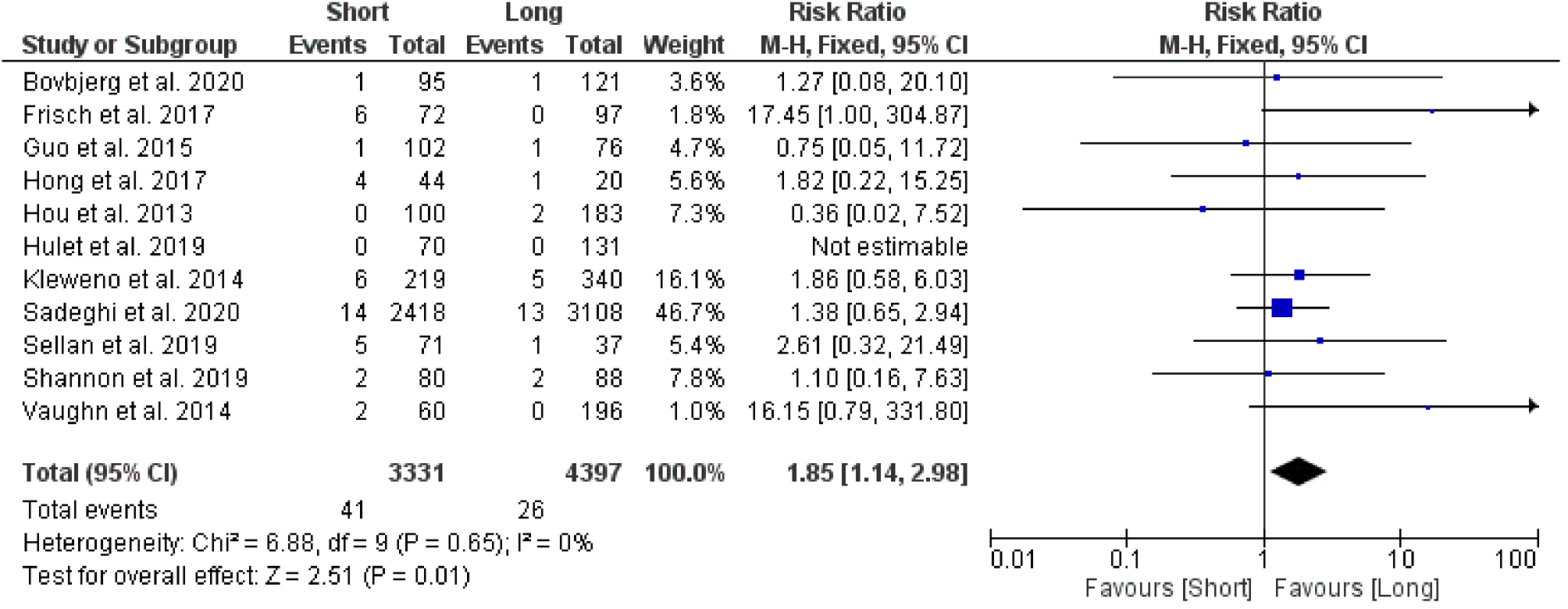
Forest plot of risk ratio for peri-implant fractures between long and short CMD groups. Blue boxes represent weighted mean values of each study. The black diamond represents the overall pooled weighted mean of all included studies.

A total of 10 studies were used to analyse the overall risk ratio for peri-implant fractures between the two groups, with results showing a statistically significant difference, favouring the group with long CMDs. The risk ratio of peri-implant fractures among patients with short CMDs was 1.85 (95% CI 1.14–2.98; *p* value = 0.01) times as likely as the risk among patients who had long CMDs (Fig. 6).

#### Reoperation rate

**Figure 7:**
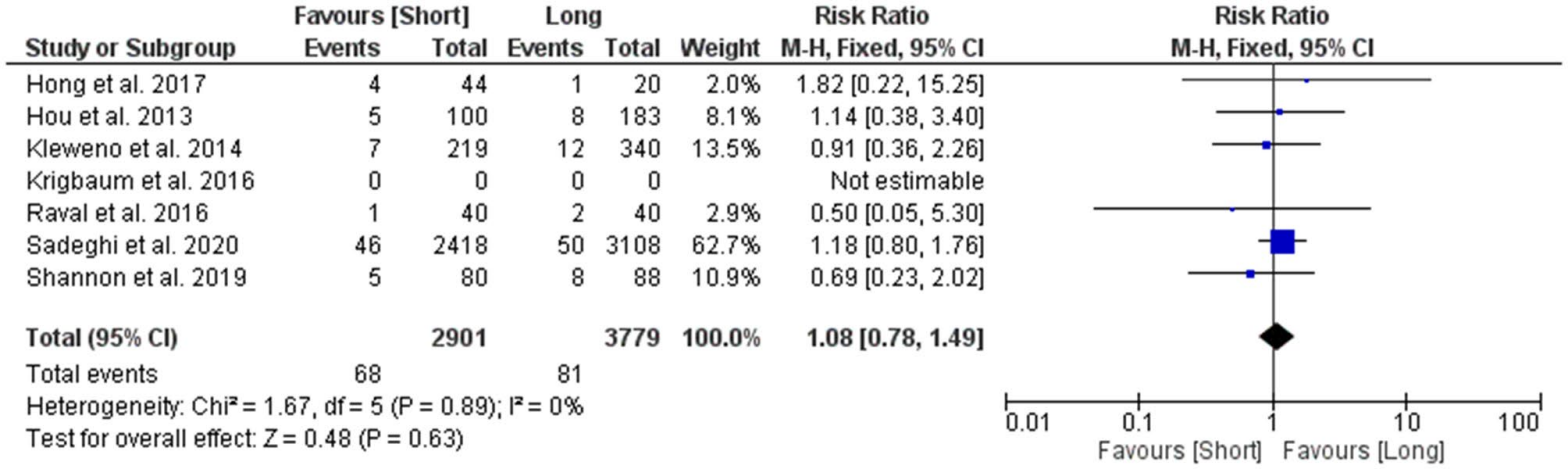
Forest plot of risk ratio for reoperation rates between long and short CMD groups. Blue boxes represent weighted mean values of each study. The black diamond represents the overall pooled weighted mean of all included studies.

A total of 6 studies were used to analyse the risk ratio of reoperation rate among patients, with results showing no statistically significant differences between the groups. The risk ratio of peri-implant fractures among patients with short CMDs was 1.08 (95% CI 0.78–1.49; *p* value = 0.63) times as likely as the risk among patients who had long CMDs (Fig. 7).

#### 1-Year Mortality Rate

**Figure 8:**
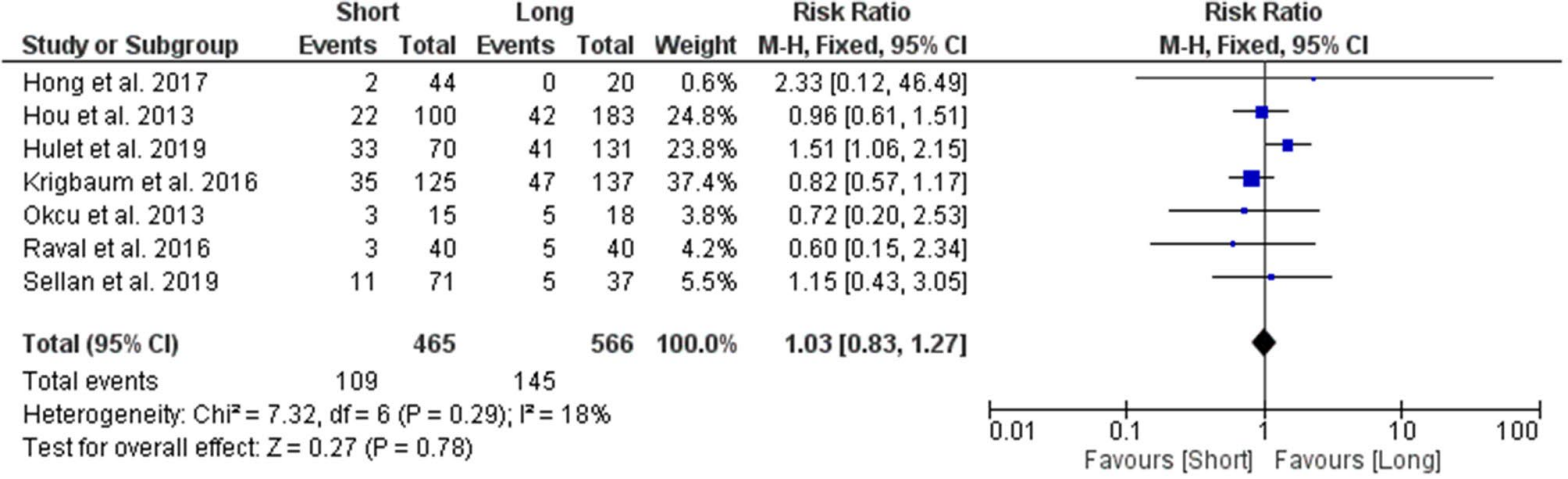
Forest plot of risk ratio for 1-year mortality rate between long and short CMD groups. Blue boxes represent weighted mean values of each study. The black diamond represents the overall pooled weighted mean of all included studies.

A total of 7 studies were used to analyse the risk ratio for 1-year mortality rate between the two groups, with results showing no statistically significant differences between the groups. The risk ratio of 1-year mortality rate among patients with long CMDs was 1.03 (95% CI 0.83–1.27; *p* value = 0.78) times as likely as the risk among patients who had short CMDs (Fig. 8).

## Discussion

The aim of the study was to evaluate differences in clinical outcomes between using short and long CMDs in the treatment of extracapsular hip fractures. With the current lack of pooled analysis and comparison between these CMDs, it is difficult for surgeons to decide with conviction the optimal nail length for their patients. Through this systematic review and meta-analyses, we have found that long and short CMDs have different short-term and long-term outcomes. Short CMDs offered significant advantages in some of the peri-operative outcomes over the long CMDs. These were shorter operative times and less blood loss. However, long CMDs have shown advantages over short CMDs in their long-term benefits. With a significant reduction in risk of peri-implant fracture rate, the longer survivorship of the prosthesis could be a reflection of the theoretical increased stability provided by having a long CMD (Table 4).

**Table 4:** Summary of findings.

Long compared to short cephalomedullary devices for fixation of extracapsular hip fractures					
Outcomes	№ of participants (studies) Follow-up	Certainty of the evidence (GRADE)	Relative effect (95% CI)	Anticipated absolute effects	
				Risk with Short cephalomedullary devices	Risk difference with long cephalomedullary devices
Operating Time	7619 (12 observational studies)	⨁⨁⨁◯ Moderate^a^	–	–	**Mean 13.99 min more** (12.84 more to 15.15 more)
Operating Time	276 (2 RCTs)	⨁⨁◯◯ Low^a,c,h^	–	–	–
Estimated Blood Loss	6593 (8 observational studies)	⨁⨁◯◯ Low^a,d^	–	–	Mean **28.81 ml higher** (23.76 higher to 33.86 higher)
Estimated Blood Loss	150 (1 RCT)	⨁⨁◯◯ Low ^e,h^	–	The mean estimated Blood Loss was 7**0** ml	Mean 18 ml** more**
Length of Stay	6594 (8 observational studies)	⨁⨁◯◯ Low^a,f^	–	–	Mean **0.13 days more** (0.09 fewer to 0.35 more)
Length of Stay	141 (2 RCTs)	⨁⨁◯◯ Low^a,g,h^	–	–	–
Peri-Implant Fractures	7452 (9 observational studies)	⨁⨁⨁◯ Moderate^a^	**RR 0.51** (0.30–0.86)	11 per 1000	**5 Fewer per 1000** (7 fewer to 1 fewer)
Peri-Implant Fractures	276 (2 RCTs)	⨁⨁◯◯ Low^a,h^	**RR 0.53** (0.14–2.00)	46 per 1000	**22 Fewer per 1000** (40 fewer to 46 more)
Reoperation Rates	6512 (6 observational studies)	⨁⨁◯◯ Low^a,b,f^	**RR 0.89** (0.78–1.49)	22 per 1000	**2 Fewer per 1000** (8 fewer to 5 more)
Reoperation Rates	168 (1 RCT)	⨁⨁◯◯ Low^h^	**RR 1.45** (0.50–4.26)	63 per 1000	**28 More per 1000** (31 fewer to 204 more)
1-Year Mortality	890 (5 observational studies)	⨁⨁◯◯ Low^a,f^	**RR 1.13** (0.83–1.27)	251 per 1000	**33 More per 1000** (28 fewer to 108 more)
1-Year Mortality	141 (2 RCTs)	⨁⨁◯◯ Low^a,h^	**RR 1.12** (0.53–2.34)	163 per 1000	**20 More per 1000** (77 fewer to 218 more)
*The risk in the intervention group (and its 95% confidence interval) is based on the assumed risk in the comparison group and the **relative effect** of the intervention (and its 95% CI) **CI:** confidence interval; **RR:** risk ratio					
**GRADE Working Group grades of evidence** **High certainty:** we are very confident that the true effect lies close to that of the estimate of the effect **Moderate certainty:** we are moderately confident in the effect estimate: the true effect is likely to be close to the estimate of the effect, but there is a possibility that it is substantially different **Low certainty:** our confidence in the effect estimate is limited: the true effect may be substantially different from the estimate of the effect **Very low certainty:** we have very little confidence in the effect estimate: the true effect is likely to be substantially different from the estimate of effect					

^a^Information used to generate the estimated effect obtained from studies with moderate risk of bias.

^b^Information used to generate the estimated effect obtained from studies with severe risk of bias.

^c^Mean operating time was reported without statistical analysis or standard deviation provided in the studies included.

^d^Estimated blood loss across studies had a large variations and standard deviation values.

^e^Estimated blood loss was reported without statistical analysis or standard deviation provided in the study included.

^f^Large amount of heterogeneity within results with results approaching the line of no effect.

^g^Length of Stay was reported without statistical analysis or standard deviation provided in the studies included.

^h^Small number of studies included for analysis.

Mean operating time between the two groups showed a significantly shorter operating time when using a short CMD as compared to a long CMD. This finding is in agreement with what has been reported in the existing literature where the number of steps and their complexity is lower when using a short CMD. This has been attributed to the additional time needed for reaming, as well the freehand placement of distal interlocking screws when inserting long CMDs^36,37^.

Estimated blood loss between the groups favoured the use of short CMDs over long CMDs, with the pooled data showing a statistically significant difference between the two groups. This has also been reported in the existing literature, with short CMDs showing a consistently lower blood loss over long CMDs^36,38^. The lower blood loss may also confer more advantages towards the use of a short nail, such as a reduction in the transfusion requirements, which had also been reported in 2 of the studies included^25,26^.

The short CMD group displayed a shorter LOS post-operatively as compared to the long CMD group. While this difference was not statistically significant, the difference in the length of stay could be due to a multitude of factors involving the condition and care of the patient. This may include the availability of community healthcare resources for the patient to be discharged to, rehabilitation services such as physiotherapy or other patient specific factors which may have altered their LOS. Another postulation is that patients who required long CMDs in the studies could have had a more complex or unstable fracture pattern which could have delayed their post-operative rehabilitation.

Rates of peri-implant fractures were significantly higher in the short CMD group, with a risk ratio of 1.85 times as compared to the long CMD group. The differences in these results may be due to the nail spanning the full length of the femur, and therefore providing additional stability and strength to the bone^16,17^. Peri-implant fractures were reported to be at the distal tip of the implant in several cases of long nails^18,23,33^. In some reports, the peri-implant fractures were due to identifiable incidents of trauma, such as falls from standing height^12,23,25,32,33^. Kleweno et al*.* reported no significant difference in the time to peri-implant fracture between the short and long nail cohorts^23^. In the studies included, the range of incidence for post-operative peri-implant fracture rates was reported to be from as early as 21 days post-operatively, up to 563 days post-operatively. It has been proposed that the timing of peri-implant fracture rates is not predictable as there is a large range of timings of reported incidence of this complication^24^.

Reoperation rates were similar in both groups, with no significant differences calculated. While the rate of peri-implant fractures 1-year post-operatively was higher in the short CMD group, rates of other complications was not well reported and thus a pooled analysis for the overall complication rate was not possible.

Finally, the 1-year mortality rate between the groups was found to be similar, with no significant difference between them. While the 1-year mortality rate has been reported to be as high as up to 58%, the current study has found significantly lower mortality rates across the groups^39^. The included studies have a 1-year mortality rate ranging from 0 (0/20) to 47% (33/70), with the vast majority reporting rates of under 30%. The decrease in 1-year mortality rate could reflect an improvement and development in operative technique, and post-operative care of hip fracture patients, thereby improving the prognosis.

Most studies included in this study have been deemed to have low levels of bias. However, those papers which have been identified to have potential issues with bias are commonly due to confounding variables, which most commonly are due to a significant difference between the ages of the short and long CMD groups. The studies which had these differences postulated that the difference could be attributed to shorter operative time which would be favourable in older age groups^12,25,26^.

In light of our analysis, we postulate that the use of short CMDs may be better suited for high-risk patients who may not be able to tolerate longer operating times. This includes those who have multiple medical comorbidities, and the elderly. The decrease in the amount of time under anaesthesia could be advantageous for these patients as it may decrease the risk of perioperative complications^13,40^. However, this should also be weighed against benefit in long-term outcomes that have been shown with the use of a long CMD.

While the study has presented several findings that could help guide the decision between the use of a short or long CMD, the strength of this study is in the volume of patients included in the analysis. This is the largest meta-analysis on the topic thus far, with the inclusion of large multi-centre data in the pooled analysis. Limitations of the paper would include the lack of RCTs included. While there were 3 included in the analysis, a larger pool of RCTs would be helpful in ascertaining the differences between short and long CMDs. Additionally, there was limited data available for some of the outcomes of interest, including 1-year post-operative HHS and transfusion rate, preventing a pooled analysis on these outcomes. Furthermore, the mean follow-up period of the included studies was varied (10.2–40.6 months), making a cross sectional study of the outcomes of interest difficult. While a key point of interest would be to investigate incidence and timeline of peri-implant fracture rates, few studies reported the time to the incidence of peri-implant fracture. This precluded further analysis on this subject, and the current study is only able to draw a conclusion to the overall rate of complications and peri-implant fractures. Other potentially interesting areas of further research on this topic would include a comparison between the peri-implant fracture rate in different periods post-operatively, including short- and long-term studies on the topic.

## Conclusion

Short CMDs offer advantages of shorter operative time and lesser blood loss. However long CMDs could offer longer-term protection against peri-implant fractures. Therefore, in planning for the operation, a patient specific approach may be necessary to make a decision according to the individual risk profile of the patient.

## Supplementary Information

Supplementary Information 1.

Supplementary Information 2.

Supplementary Information 3.

Supplementary Information 4.
