# Supplementary material for: Clinical outcomes following long versus short cephalomedullary devices for fixation of extracapsular hip fractures: a systematic review and meta-analysis
Source: Sci Rep. 2021 Dec 14;11:23997. doi: 10.1038/s41598-021-03210-1 (PMC8671534; doi:10.1038/s41598-021-03210-1)
Supplement: Supplementary file 1 — Supplementary Information 1. [file 41598_2021_3210_MOESM1_ESM.docx]

Table 1 GRADE evidence profile for outcomes assessed

| **Certainty assessment** | | | | | | | **№ of patients** | | **Effect** | | **Certainty** | **Importance** |
| --- | --- | --- | --- | --- | --- | --- | --- | --- | --- | --- | --- | --- |
| **№ of studies** | **Study design** | **Risk of bias** | **Inconsistency** | **Indirectness** | **Imprecision** | **Other considerations** | **Long** | **short cephalomedullary devices** | **Relative (95% CI)** | **Absolute (95% CI)** |  |  |
| **Operating Time** | | | | | | | | | | | | |
| 12 | observational studies | serious^a^ | not serious | not serious | not serious | none | 4234 | 3385 | - | mean **13.99 minutes more** (12.84 more to 15.15 more) | ⨁⨁⨁◯ Moderate | IMPORTANT |
| **Operating Time** | | | | | | | | | | | | |
| 2 | randomised trials | serious^a^ | not serious | not serious | Serious^c^ | none | 125 | 151 | - | - | ⨁⨁◯◯ Low | IMPORTANT |
| **Estimated Blood Loss** | | | | | | | | | | | | |
| 8 | observational studies | serious^a^ | not serious | not serious | serious^d^ | none | 3682 | 2911 | - | mean **28.81 ml higher** (23.76 higher to 33.86 higher) | ⨁⨁◯◯ Low | IMPORTANT |
| **Estimated Blood Loss** | | | | | | | | | | | | |
| 1 | randomised trials | not serious | not serious | not serious | Serious^e, h^ | none | 80 | 70 | - | - | ⨁⨁◯◯ Low | IMPORTANT |
| **Length of Stay** | | | | | | | | | | | | |
| 8 | observational studies | serious^a^ | Serious^f^ | not serious | not serious | none | 3683 | 2911 | - | mean **0.13 days more** (0.09 fewer to 0.35 more) | ⨁⨁◯◯ Low | IMPORTANT |
| **Length of Stay** | | | | | | | | | | | | |
| 2 | randomised trials | serious^a^ | not serious | not serious | Serious^g, h^ | none | 55 | 86 | - | - | ⨁⨁◯◯ Low | IMPORTANT |
| **Peri-Implant Fractures** | | | | | | | | | | | | |
| 9 | observational studies | serious^a^ | not serious | not serious | not serious | none | 23/4272 (0.5%) | 34/3180 (1.1%) | **RR 0.51**  **(0.30 to 0.86)** | **5 fewer per 1,000**  **(from 7 fewer to 1 fewer)** | ⨁⨁⨁◯ Moderate | CRITICAL |
| **Peri-Implant Fractures** | | | | | | | | | | | | |
| 2 | randomised trials | serious^a^ | not serious | not serious | not serious^h^ | none | 3/125 (2.4%) | 7/151 (4.6%) | **RR 0.53** (0.14 to 2.00) | **22 fewer per 1,000** (from 40 fewer to 46 more) | ⨁⨁◯◯ Low | CRITICAL |
| **Reoperation Rates** | | | | | | | | | | | | |
| 6 | observational studies | serious^a,b^ | Serious^f^ | not serious | not serious | none | 73/3691 (2.0%) | 63/2821 (2.2%) | **RR 0.89**  **(0.64 to 1.24)** | **2 fewer per 1,000**  **(from 8 fewer to 5 more)** | ⨁⨁◯◯ Low | CRITICAL |
| **Reoperation Rates** | | | | | | | | | | | | |
| 1 | randomised trials | not serious | not serious | not serious | serious^h^ | none | 8/88 (9.1%) | 5/80 (6.3%) | **RR 1.45** (0.50 to 4.26) | **28 more per 1,000** (from 31 fewer to 204 more) | ⨁⨁◯◯ Low | CRITICAL |
| **1-Year Mortality** | | | | | | | | | | | | |
| 5 | observational studies | serious^a^ | Serious^f^ | not serious | not serious | none | 135/511 (26.4%) | 95/379 (25.1%) | **RR 1.13**  **(0.89 to 1.43)** | **33 more per 1,000**  **(from 28 fewer to 108 more)** | ⨁⨁◯◯ Low | CRITICAL |
| **1-Year Mortality** | | | | | | | | | | | | |
| 2 | randomised trials | serious^a^ | not serious | not serious | not serious^h^ | none | 10/55 (18.2%) | 14/86 (16.3%) | **RR 1.12** (0.53 to 2.34) | **20 more per 1,000** (from 77 fewer to 218 more) | ⨁⨁◯◯ Low | CRITICAL |

**CI:** confidence interval; **RR:** risk ratio

#### Explanations

a. Information used to generate the estimated effect obtained from studies with moderate risk of bias

b. Information used to generate the estimated effect obtained from studies with severe risk of bias

c. Mean operating time was reported without statistical analysis or standard deviation provided in the studies included

d. Estimated blood loss across studies had a large variations and standard deviation values

e. Estimated blood loss was reported without statistical analysis or standard deviation provided in the study included

f. Large amount of heterogeneity within results with results approaching the line of no effect

g. Length of Stay was reported without statistical analysis or standard deviation provided in the studies included

h. Small number of studies included for analysis
