# Supplementary material for: Clinical outcomes following long versus short cephalomedullary devices for fixation of extracapsular hip fractures: a systematic review and meta-analysis
Source: Sci Rep. 2021 Dec 14;11:23997. doi: 10.1038/s41598-021-03210-1 (PMC8671534; doi:10.1038/s41598-021-03210-1)
Supplement: Supplementary file 2 — Supplementary Information 2. [file 41598_2021_3210_MOESM2_ESM.docx]

Table 2 Risk assessment of randomized controlled trials with the revised Cochrane risk-of-bias tool (RoB 2)

| Study | Randomization Process | Deviations from the intended interventions | Outcome | Missing outcome data | Measurement of the outcome | Selection of the reported result | Overall |
| --- | --- | --- | --- | --- | --- | --- | --- |
| Sellan et al (23) | High Risk | Some Concerns | Operating Time | Low Risk | Low Risk | Low Risk | Some Concerns |
|  |  |  | Estimated Blood Loss | Low Risk | Low Risk | Low Risk | Some Concerns |
|  |  |  | Length of Stay | Low Risk | Low Risk | Low Risk | Some Concerns |
|  |  |  | Peri-Implant Fracture | Low Risk | Low Risk | Low Risk | Some Concerns |
|  |  |  | 1-Year Mortality | Low Risk | Low Risk | Low Risk | Some Concerns |
| Okcu et al (24) | Some Concerns | Some Concerns | Mean Operating Time | Low Risk | Some Concerns | Low Risk | Some Concerns |
|  |  |  | Length of Stay | Low Risk | Some Concerns | Low Risk | Some Concerns |
|  |  |  | Reoperation Rates | Low Risk | Some Concerns | Low Risk | Some Concerns |
|  |  |  | 1-Year Mortality | Low Risk | Some Concerns | Low Risk | Some Concerns |
| Shannon et al (25) | Low Risk | High Risk | Operating Time | Some Concerns | Low Risk | Low Risk | Some Concerns |
|  |  |  | Estimated Blood Loss | Some Concerns | Low Risk | Low Risk | Some Concerns |
|  |  |  | Reoperation Rates | Some Concerns | Low Risk | Low Risk | Some Concerns |
|  |  |  | Peri-Implant Fracture | Some Concerns | Low Risk | Low Risk | Some Concerns |
