# Supplementary material for: Clinical outcomes following long versus short cephalomedullary devices for fixation of extracapsular hip fractures: a systematic review and meta-analysis
Source: Sci Rep. 2021 Dec 14;11:23997. doi: 10.1038/s41598-021-03210-1 (PMC8671534; doi:10.1038/s41598-021-03210-1)
Supplement: Supplementary file 3 — Supplementary Information 3. [file 41598_2021_3210_MOESM3_ESM.docx]

Table 3 Risk assessment of non-randomized studies with the risk-of-bias in non-randomized studies – of interventions (ROBINS-I) tool

| Study | Bias due to confounding | Bias in selection of participants into the study | Bias in classification of interventions | Outcome | Bias due to deviations from intended interventions | Bias due to missing data | Bias in measurement of outcomes | Bias in selection of the reported result | Overall |
| --- | --- | --- | --- | --- | --- | --- | --- | --- | --- |
| Hulet et al. (34) | Low | Moderate | Moderate | 1-Year Mortality | Low | Low | Low | Low | Moderate |
| Krigbaum et al. (32) | Moderate | Moderate | Low | Operating Time | Low | Low | Low | Low | Moderate |
|  |  |  |  | Length of Stay | Low | Low | Low | Low | Moderate |
|  |  |  |  | Reoperation Rate | Low | Low | Low | Low | Moderate |
|  |  |  |  | 1-Year Mortality | Low | Low | Low | Low | Moderate |
| Klewno et al. (26) | Serious | Moderate | Moderate | Operating Time | Low | Serious | Low | Low | Serious |
|  |  |  |  | Reoperation Rate | Low | Serious | Low | Low | Serious |
|  |  |  |  | Peri-Implant Fracture | Low | Serious | Low | Low | Serious |
| Hou et al. (18) | Low | Low | Moderate | Operating Time | Low | Low | Low | Low | Moderate |
|  |  |  |  | Estimated Blood Loss | Low | Low | Low | Low | Moderate |
|  |  |  |  | Length of Stay | Low | Low | Low | Low | Moderate |
|  |  |  |  | Reoperation Rate | Low | Low | Low | Low | Moderate |
|  |  |  |  | Peri-Implant Fracture | Low | Low | Low | Low | Moderate |
|  |  |  |  | 1-Year Mortality | Low | Low | Low | Low | Moderate |
| Frisch et al. (31) | Low | Low | Low | Operating Time | Low | Low | Low | Low | Low |
|  |  |  |  | Estimated Blood Loss | Low | Low | Low | Low | Low |
|  |  |  |  | Peri-Implant Fracture | Low | Low | Low | Low | Low |
| Vaughn et al. (12) | Serious | Low | Moderate | Peri-Implant Fracture | Moderate | Low | Low | Low | Serious |
| Boone et al. (27) | Serious | Low | Serious | Operating Time | Moderate | Low | Low | Low | Serious |
|  |  |  |  | Estimated Blood Loss | Moderate | Low | Low | Low | Serious |
|  |  |  |  | Length of Stay | Moderate | Low | Low | Low | Serious |
|  |  |  |  | Peri-Implant Fracture | Moderate | Low | Low | Low | Serious |
| Guo et al. (28) | Moderate | Low | Moderate | Operating Time | Low | Low | Low | Low | Moderate |
|  |  |  |  | Estimated Blood Loss | Low | Low | Low | Low | Moderate |
|  |  |  |  | Length of Stay | Low | Low | Low | Low | Moderate |
|  |  |  |  | Peri-Implant Fracture | Low | Low | Low | Low | Moderate |
| Hong et al. (29) | Low | Low | Low | Operating Time | Low | Low | Low | Low | Low |
|  |  |  |  | Length of Stay | Low | Low | Low | Low | Low |
|  |  |  |  | Reoperation Rate | Low | Low | Low | Low | Low |
|  |  |  |  | Peri-Implant Fracture | Low | Low | Low | Low | Low |
|  |  |  |  | 1-Year Mortality | Low | Low | Low | Low | Low |
| Sadeghi et al. (20) | Low | Low | Low | Operating Time | Low | Low | Low | Low | Low |
|  |  |  |  | Estimated Blood Loss | Low | Low | Low | Low | Low |
|  |  |  |  | Length of Stay | Low | Low | Low | Low | Low |
|  |  |  |  | Reoperation Rate | Low | Low | Low | Low | Low |
|  |  |  |  | Peri-Implant Fracture | Low | Low | Low | Low | Low |
| Li et al. (30) | Low | Low | Low | Operating Time | Low | Moderate | Moderate | Low | Moderate |
|  |  |  |  | Estimated Blood Loss | Low | Moderate | Moderate | Low | Moderate |
|  |  |  |  | Length of Stay | Low | Moderate | Moderate | Low | Moderate |
| Raval et al. (33) | Low | Low | Moderate | Operating Time | Low | Low | Low | Low | Moderate |
|  |  |  |  | Estimated Blood Loss | Low | Low | Low | Low | Moderate |
|  |  |  |  | Length of Stay | Low | Low | Low | Low | Moderate |
|  |  |  |  | Reoperation Rate | Low | Low | Low | Low | Moderate |
|  |  |  |  | 1-Year Mortality | Low | Low | Low | Low | Moderate |
| Bovbjerg et al. (35) | Low | Low | Low | Peri-Implant Fracture | Low | Moderate | Low | Low | Moderate |
