# Supplementary material for: Clinical outcomes following long versus short cephalomedullary devices for fixation of extracapsular hip fractures: a systematic review and meta-analysis
Source: Sci Rep. 2021 Dec 14;11:23997. doi: 10.1038/s41598-021-03210-1 (PMC8671534; doi:10.1038/s41598-021-03210-1)
Supplement: Supplementary file 4 — Supplementary Information 4. [file 41598_2021_3210_MOESM4_ESM.docx]

**Search Strategy**

1. Hip Fractures.mp. or exp Hip Fractures/

2. Intertrochanteric Fractures.mp.

3. (pertrochanteric fracture or pertrochanteric fractures).mp.

4. 1 or 2 or 3

5. Fracture Fixation, Intramedullary.mp. or exp Fracture Fixation, Intramedullary/

6. (cephalomedullary nail* or cephalo medullary nail*).mp.

7. intermedullary nail*.mp.

8. 5 or 6 or 7

9. 4 and 8
